# Hymenoptera Venom Allergy: How Does Venom Immunotherapy Prevent Anaphylaxis From Bee and Wasp Stings?

**DOI:** 10.3389/fimmu.2019.01959

**Published:** 2019-08-21

**Authors:** Umit Murat Sahiner, Stephen R. Durham

**Affiliations:** ^1^Immunomodulation and Tolerance Group, Allergy and Clinical Immunology Inflammation, Repair and Development, National Heart and Lung Institute, Imperial College London, London, United Kingdom; ^2^Pediatric Allergy Department, Hacettepe University School of Medicine, Ankara, Turkey; ^3^MRC and Asthma UK Centre in Allergic Mechanisms of Asthma, London, United Kingdom

**Keywords:** allergy, anaphylaxis, venom, immunotherapy, immune tolerance

## Abstract

Hymenoptera stings may cause both local and systemic allergic reactions and even life threatening anaphylaxis. Along with pharmaceutical drugs and foods, hymenoptera venom is one of the most common causes of anaphylaxis in humans. To date, no parameter has been identified that may predict which sensitized people will have a future systemic sting reaction (SSR), however some risk factors, such as mastocytosis and age >40 years are known. Venom immunotherapy (VIT) is the most effective method of treatment for people who had SSR, which is shown to be effective even after discontinuation of the therapy. Development of peripheral tolerance is the main mechanism during immunotherapy. It is mediated by the production of blocking IgG/IgG4 antibodies that may inhibit IgE dependent reactions through both high affinity (FcεRI) and low affinity (FcεRII) IgE receptors on mast cells, basophils and B cells. The generation of antigen specific regulatory T cells produces IL-10 and suppresses Th2 immunity and the immune responses shift toward a Th1-type response. B regulatory cells are also involved in the production of IL-10 and the development of long term immune tolerance. During VIT the number of effector cells in target organs also decreases, such as mast cells, basophils, innate type 2 lymphocytes and eosinophils. Several meta-analyses and randomized controlled studies have proved that VIT is effective for preventing SSR to a sting and improves the quality of life. In this review, the risk of SSR in venom allergy and how VIT changed this risk are discussed.

## Introduction

The Hymenoptera insect group includes Apidae and Vespidae subgroups and also the Formicidae, which is beyond the scope of this review. Apidae consists of Apis mellifera (honey bees) and Bumblebee species (bumblebees), and the Vespidae subclass includes Vespula species (“yellow jackets,” wasps and hornets) and Polistes species (“paper” wasps) (1, 2). Honeybee stings are generally not more severe but they inject more venom. Bees inject 50–140 micrograms of venom whereas wasps deliver nearly 3 μg of venom with each sting. Bees can sting once but wasps have the capacity to sting multiple times (1–3). Insect sting allergy may cause local, large local (>10 cm in diameter) or even systemic reactions (SR), and potentially life threatening anaphylactic reactions (4–6). The rate of systemic sting reactions in epidemiological studies in Europe ranged between 0.3 and 7.5% in adults (7) and 0.15–3.4% in children (7, 8). The chance of a SR and the chance of life threatening anaphylaxis are related to many factors, including the severity of the preceding reaction, allergy to bee venom, the level of baseline serum tryptase and presence of mastocytosis, increased basophil activation, age and underlying medical conditions (7).

Venom immunotherapy (VIT) leads to complete protection from SSR in 77–84% of cases for honeybee and 91–96% for vespid venoms (9–11). The frequency of systemic adverse events during VIT ranges between 8 and 20% from large multicenter studies (12, 13). In a recent study by Stoevesandt et al. a systemic reaction rate of 11.7% (any reactions including the subjective ones) was reported during build-up phase of VIT; however the SSR rate dropped to 3% when objective diagnostic criteria of anaphylaxis was used (14). The most important risk factors related to systemic reactions during VIT are honeybee venom immunotherapy, rapid dose increase during the build-up phase and probably high basal tryptase levels in vespid allergy but not in honeybee venom allergy (9). The protective effect of VIT persists for years after stopping treatment. The long term outcome of systemic reactions after discontinuation of VIT is superior in children compared to adults and for vespid venom compared to honeybee VIT (15–17).

This review aims to discuss first the epidemiology and risk factors of insect venom anaphylaxis, then focuses on the mechanisms of VIT to prevent SSR to insect stings and finally aims to discuss the efficacy, safety and long term effects of VIT as well as the risk factors related to SSR during and after VIT.

## Epidemiology of Venom Allergy and Allergic Reactions

The prevalence of being stung by Hymenoptera species during life ranges from 56.6 to 94.5% in adults and 37.5% in children up to 14 years of age (7, 8). The sensitization rate, indicated either by a positive skin prick test or by specific IgE positivity, ranges between 9.3 and 28.7% in adults. In one study children were found to be 3.7% positive to Hymenoptera species (mostly honeybee) by skin prick testing (18).

The rate of systemic sting reactions in epidemiological studies in Europe ranges between 0.3 and 7.5% in adults (7). Among these reactions, the anaphylactic shock frequency is between 0.6 and 42.8% (18–23). According to a recent position paper in adults, respiratory and cardiovascular symptoms may occur in as many as 70% of the systemic reactions (24). This wide range reflects the lack of consensus on the definition of anaphylaxis, differences in data collection techniques and variability in degree of exposure to stings in different climate conditions (7). In children, the prevalence of SR is much lower and ranges from 0.15 to 3.4% (7, 8). Additionally, of the SR in children about 60% are mild and restricted only to the skin (24).

Beekeepers are a vulnerable and high risk population for the development of allergic reactions to honeybee stings. In this specific group, the SR rates are higher than the general population and range from 14 to 38% (25). Receiving more than 200 stings per year is nearly totally protective from a SR whereas receiving fewer than 25 stings per year is related to a SR rate of 45% (25). In a British beekeeper survey, risk factors for SR were found to be female gender, positive family history of bee venom allergy, premedication with antihistamines before hive attendance and 2 or more years of beekeeping before the first SR (26).

According to the European network of severe allergic reactions (NORA), 20.2% of all the anaphylaxis cases in children and 48.2% of documented anaphylaxis in adults were due to insect venom. In this study, 59 tertiary allergy, dermatology and pediatrics centers from 10 different countries reported 3,333 anaphylaxis cases (27). Only 27.6% of insect anaphylaxis cases received on-site adrenaline (27). In population based studies performed during the first decade of the twenty-first century, the rate of anaphylaxis due to insect venom ranged from 7.3 to 59% and was found mostly in adults (7).

Fatalities from insect stings have been examined previously in a number of studies. A study from Costa Rica reported 52 deaths in a 22 years period accounting to 0.74 deaths per million inhabitants per year (28), which is much higher than a study from the USA with a number of 0.14 deaths per million inhabitants per year (29). A recent report from the UK stated 93 deaths from venom anaphylaxis between 1992 and 2012, accounting to 0.09 deaths per million inhabitants per year (30). Especially a previous history of hymenoptera allergy, male sex, older age and delayed adrenaline administration are important risk factors for fatal reactions (31, 32).

Risk factors related to SSR to hymenoptera stings are summarized in Table 1.

**Table 1:** Risk factors for severe systemic reactions/anaphylaxis to hymenoptera stings.

**Risk factor**	**Characteristics**
Age around >40 years (33, 34)	Milder reactions in children, higher risk in adults especially age over 40 years
Elevated basal serum tryptase (34, 35)	In patients even without mastocytosis there is an increased risk of severe systemic reactions
Mastocytosis (35–37)	Especially in adult patients with mastocytosis and in indolent mastocytosis without skin lesions
Absence of skin smptoms during anaphylaxis (34, 38)	Lack of urticaria and angioedema may be related to the indolent systemic mastocytosis without skin lesions
Short time interval between sting and onset of symptoms (34, 38)	If symptoms start in <5 min, risk of severe systemic reaction increases
Severity of the previous reaction (7)	The more severe the previous reaction, the greater the risk of a future severe reaction
Angiotensin converting enzyme inhibitor and β-blockers usage (Cardiac comorbidities) (14, 39)	Debated. May increase the reaction severity not the reaction risk

## Venom Allergy, Mast Cell Activation Disorders and Anaphylaxis

Mastocytosis is a clonal neoplastic disorder of mast cells that is characterized primarily by cutaneous or systemic subtypes and sometimes by rare forms. Mastocytosis usually involves the somatic KIT D816V mutation, shows aberrant CD25 or CD2 expression on mast cells and is frequently accompanied by elevated baseline serum tryptase (BST) (40). In patients with cutaneous mastocytosis, mast cell aggregates are located only in the skin and this form is mostly diagnosed in infants and children (41). Systemic mastocytosis (SM) is frequently found in adults and is divided into four subtypes (indolent SM, SM associated with a hematological disorder, aggressive SM and mast cell sarcoma); the most common among these is indolent systemic mastocytosis (ISM). ISM can present with normal BST and lack of skin lesions (42). The prevalence of mastocytosis in patients with hymenoptera venom allergy may be as high as 7.9%, which is significantly higher than that of the general population (3–13/100,000 inhabitants) (43, 44). Similarly, hymenoptera stings are the most common cause of anaphylaxis in people with mastocytosis, and the prevalence is nearly 30% (45). In a considerable number of patients with ISM, hymenoptera venom anaphylaxis (HVA) may be the first symptom of the disease. These ISM patients without skin lesions are mostly males with lower BST levels and lower frequency of KIT mutations (46). Anaphylaxis in patients with mastocytosis is mostly characterized by cardiac symptoms, such as hypotension and syncopal episodes, whereas skin symptoms, such as urticaria and angioedema are rare. Therefore, if a patient with VHA presents with hypotension and syncope without skin symptoms, mastocytosis should be suspected (47). Recently, for the cases of HVA, a scoring system called REMA was developed based on gender, clinical symptoms and BST to identify patients having clonal mast cell systemic disease. A score of ≥2 suggests that ISM should be ruled out with a sensitivity and specificity of 92 and 81%, respectively (47).

The precise mechanisms for the increased prevelance of HVA in mastocytosis are not well-understood. However, possible explanations for this co-existence are as follows: (1) Increased mast cell burden may lead to higher mediator release in the case of a sting; (2) The perivascular location of the mast cells may cause a direct access of mediators to the systemic circulation, which increase the severity of the reaction; (3) A gain of function mutation, KIT D816V, may prevent mast cell apoptosis and increases the proliferation and differentiation of the mast cells, and finally (4) Phospholipase A2, a component of hymenoptera venom may cause a direct mediator release from mast cells (36).

VIT seems to be the most appropriate treatment option for patients with mastocytosis and HVA. VIT is usually well-tolerated, safe and effective. Whether BST increases the risk of adverse events during VIT is not yet clear. Despite the preference for lifelong VIT in patients with mastocytosis, there is no evidence based proof of lifelong treatment in the literature. However, to be on the safe side it is recommended that VIT should be prolonged in patients with elevated SBT (>11.4 μg/L) or verified mastocytosis if the initial SSR was severe (9).

## Mechanisms of Venom Immunotherapy

**Figure 1:**
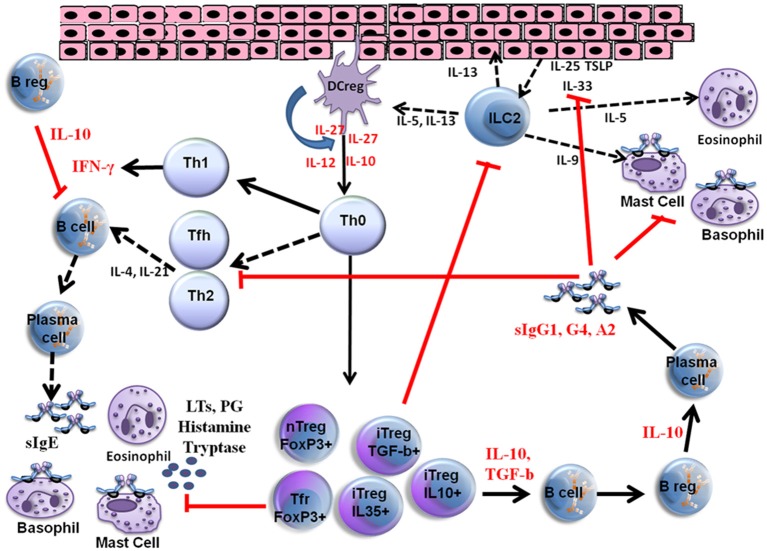
Mechanisms of venom allergen immunotherapy. High dose of hymenoptera venom stimulates dendritic cells and induces Treg and B reg cells as well as other B cell subsets that produce allergen specific IgG1, IgG4, and IgA type bloking antibodies. Several cytokines also take place in the immune tolerance induction and as a result a shift from Th2 to Th1 type immune deviation occurs. Red arrows show blocking activity induced during VIT. LTs, Leukotrienes; PG, Prostaglandins; iTreg, Inducible Tregulatory cells; nTreg, Natural T regulatory cells; Tfh, T follicular hepler cells; Tfr, T Follicular regulatory cells; DC reg, Regulatory dendritic cells; TSLP, Thymic stromal Lymphopoietin.

VIT is the only effective and immune modulatory treatment in patients with a history of anaphylaxis and has proven to be effective for between 80 and 95% of patients with bee venom and vespula venom allergy, respectively (9–11). Initially, the interaction between allergens, epithelial cells and dendritic cells triggers the response produced by the innate immune system and subsequently the adaptive immune responses develop. Different parts of the immune system are involved in the development of peripheral immune tolerance, and in a network of different cell types, either directly through cell to cell contact or through release of various cytokines and specific antibody production (IgE, IgG1, IgG4, IgA). Further details of VIT mechanisms are shown in Figure 1.

### Allergen Specific Antibody Responses

Initially subcutaneous VIT is associated with transient early increases in serum allergen specific IgE (sIgE) levels and then there is a decrease in sIgE over several years (48–50). AIT is also associated with increases in allergen-specific IgA, IgG1 and IgG4 antibodies, which are called blocking antibodies (48, 51). Studies with aeroallergens have shown significant increases in serum concentrations of blocking antibodies, up to 100 times in a time and dose dependent manner (52–54). The production of blocking antibodies, particularly the IgG4 type, can compete with sIgE for allergen and prevent the allergen-sIgE interaction. The blockage of allergen-sIgE interactions prevents cross-linking of high-affinity IgE receptors (FcεRI) on basophils and mast cells, which inhibits degranulation of these cells and may prevent the development of anaphylaxis (55, 56). Blocking antibodies, IgG/IgG4, inhibit the IgE-facilitated allergen presentation to T cells by blocking low-affinity receptors (FcgRIIb) on B cells and stop the allergen induced boost of memory IgE production (54, 57, 58). One of the major cytokines produced by T regs, IL-10, not only is involved in the suppression of allergen-specific T effector cells during AIT, but it also inhibits the production of total IgE and specific IgE, while increasing IgG4 levels (59, 60). In non-allergic beekeepers, the IgG4 serum concentrations are nearly one thousand times higher than the serum specific IgE levels. The serum IgG4 concentrations are closely correlated with the number stings per year and time spent in beekeeping (31, 61). In an animal model of bee venom allergy, major bee venom allergen PLA2 was injected into inguinal lymph nodes and an allergen-specific IgG response with the production of IgG2a was observed (62). In another study of peptide VIT, a reduction in allergen-specific IgE and an increase in specific IgG2a were found, both of which had preventive functions against allergen-induced anaphylaxis (63).

After stopping AIT for grass pollen allergy, it was shown that blocking IgG/IgG4 levels decreased nearly 80–90% within 1 year. In contrast, IgG-associated serum IgE-inhibitory activity persisted for several years and correlated with clinical efficacy (64). This suggests that the functional activity of blocking antibodies rather than their levels may be a more accurate measure of clinical efficacy and seems to correlate closely with long term immune tolarance (54). However, this may not be the case for bee venom immunotherapy, where although successful desensitization was accompanied by increases in both IgG4 and IgE-Inhibitory activity, both the elevated specific IgG4 levels and IgE-FAB inhibitory activity returned to baseline within months of discontinuation of VIT and further follow up revealed a more sustained decrease in venom-specific IgE levels (65) representing a putative alternative mechanism of prolonged protection following IgG withdrawal. This is also supported by the observation of low/absent IgE levels in tolerant beekeepers (66).

### Effector Cell Responses

VIT shows its action through complex immunological mechanisms. The initial mechanism of action seen on effector cells is mast cell and basophil desensitization (9). The number of these cells decreases during venom immunotherapy and additionally their thresholds for cytokine release increase with time. During the rush VIT early in the course of treatment there is a decrease in peripheral blood basophil numbers and also in the production and release of basophil derived cytokines, such as IL-4 and IL-13 (67). Basal serum tryptase level, which is a marker of mast cell burden and mast cell function, decreases over time during VIT (68). In patients with VIT, the suppression of surface antigens on blood basophils was shown previously (69). In addition to the changes observed in basophil surface antigens, the amount of histamine released from basophils following sting challenges also decreased in patients with VIT depending on their clinical reactivity (70). Basophil reactivity assessed in the flow-cytometric evaluation of CD63 expression has been shown to be a reliable diagnostic test to diagnose hymenoptera venom allergy (71). Basophil sensitivity, the dose at which half of the maximum basophil response occurs, was suggested to monitor VIT (72–74). A new method of functional assay that measures intracellular staining of phycoerythrin-conjugated daimine oxidase (DAO) has been validated for detecting the amount of histamine released from basophils. Following allergen stimulation, intracellular DAO levels decrease in proportion to the intracellular histamine released. This reduction was shown in patients treated with vespula VIT, which is important for increasing the threshold for venom to induce an anaphylactic reaction in VIT patients (75). Not only the preformed mediator release but also the production and release of newly generated mediators, such as leukotriene C4 in blood basophils in patients following VIT decreases (76).

Following rush VIT a decrease in T-cell expressed and secreted (RANTES) protein, IL-8 and monocyte chemoattractant protein 1 (MCP-1) production have been reported in peripheral blood mononuclear cell (PBMC) cultures at protein and as well as mRNA levels (77).

During the early phases of VIT the mechanisms that start desensitization are not fully understood. In 2010 Bussmann et al. performed a study on patients with rush VIT. They analyzed expression levels of different tolerogenic markers at protein and mRNA levels within the first 5 days of VIT. They observed a prominent degradation of tryptophan, which is linked to the suppression of T cell responses and induction of tolerance; elevated ILT3 and ILT4, which are inhibitory receptors for monocytes, and IL-10 production of CD3^+^ T cells and monocytes followed by increased IL-10 serum levels, which is an important regulatory interleukin for the suppression of allergen induced responses (78).

In studies with aeroallergens, AIT was shown to inhibit early and late phase allergic responses at allergic tissue sites through the suppression of several cytokines and decreases in numbers of eosinophils, mast cells and basophils (54). This information indicates that allergen immunotherapy is effective at both systemic and local levels. Similar mechanisms are likely to apply to VIT.

### T and B Cell Responses During VIT

The development of immune tolerance during VIT has been shown to be related to the modification of T and B cell responses (79). A Th2 to Th1 shift occurs during VIT and an increase in interferon gamma (IFN-γ) levels is observed parallel to the decrease in IL-4 and IL-13 in whole blood (80, 81). Th2 responses during VIT are reduced and there is also an increase in Treg cell numbers and functions (80, 82). Treg cells are divided into 2 subgroups as natural regulatory T (nTreg) cells, which are characterized by the transcription factor forkhead box P3 (FOXP3), and inducible regulatory T (iTreg) cells, such as IL-10 producing Tr1 cells and TGF-b producing TH3 cells (83–85). IL-10 plays an inhibitory role in B cells by blocking B7/CD28 pathway. This results in a supressive effect on dendritic cell maturation and in MHC class II and costimulatory ligand expressions (86). TGF-b downregulates FceRI expression on Langerhans cells and also upregulates FOXP3 and RUNX and assists CTLA-4 expression on T cells (87, 88).

In beekeepers IL-10 producing Treg cells inhibit the proliferation of PLA-specific effector T cells shortly after the start of bee venom season. This suppressive effect can be reversed by blocking CTLA-4, PD-1, and IL-10 receptors (89). Additionally induction of indoleamine 2,3-dioxygenase enzyme in dendritic cells, by the effect of Tregs, causes the transformation of inflammatory dendritic cells into regulatory dendritic cells (90). In a similar manner, during VIT, Tr1-type Treg cell proliferation is prominent and the antigen-specific proliferative and cytokine responses against the major bee venom allergen, the phospholipase A2 (PLA) have been significantly suppressed by the end of first week of VIT (91). The allergen induced secretion of Th2 cytokines, such as IL-4, IL-5, and IL-13 were abolished (92). In addition to IL-10 production, Treg cells can also suppress immune responses via cell-to-cell interactions.

The role of increased IL-10 levels is prominent in the development of clinical and immunological tolerance during VIT. Blockage of IL-10 in PBMC reconstitutes the specific proliferative and cytokine responses. This situation can also be seen in beekeepers who have received multiple bee stings (91). The presence of increased numbers of CD4^+^CD25^+^FOXP3^+^ Treg cells in the target organ, nasal mucosa, after grass pollen allergen immunotherapy suggests that Treg cells play an important for the development of allergen-specific immune tolerance (93). In a similar manner, VIT was found to be related to the progressive expansion of circulating CD4^+^CD25^+^FOXP3^+^ Treg cell numbers (94). During all types of AIT a deviation toward a regulatory/suppressor T cell response has been reported (95). In a study by Nasser et al allergen-induced changes in cytokine mRNA and cellular profiles from cutaneous biopsies were compared before and 3 months after wasp VIT. There was a significant decrease in IL-4 mRNA and an increase in IL-10^+^ cells. Additionally a trend toward an increase in IL-10 mRNA was also observed (96). In another study by Schuerwegh et al, the effect of VIT on CD4^+^CD8^+^ T lymphocytes were evaluated before VIT, at the end of 5 days semi-rush VIT and at 6 months during VIT. A significant decrease in IL-producing CD4^+^ and CD8^+^ T cell numbers, compared with cytokine-producing cells before VIT was observed by the end of 5 days semi-rush VIT. After 6 months of VIT, a higher amount of IL-2 and IFN-γ-producing CD4^+^CD8^+^ T lymphocytes has been found confirming a shift from Th2 to Th1 type immune deviation (81). IL-10 serum levels began to increase from the second day of VIT (78) and on day 28 of treatment, a desensitized condition has arisen in allergen-specific T cells associated with the direct suppressive effects of IL-10 (94).

T follicular helper cells (T_FH_) are defined by CXCR5^+^ surface receptor and they help for B-cell maturation and immunoglobulin class-switching. CXCR5^+^ FoxP3^+^ Treg cells are a subset of Tregs, called as follicular regulatory T (T_FR_) cells, which are capable of suppressing T- and B-cell responses by migrating to germinal centers of lymph nodes (97, 98). A study by grass pollen immunotherapy has shown a significant decrease in memory T_FH_ cell numbers after immunotherapy (99). Additionally, T_FR_ cells were found to produce more IL-10 compared to T_FH_ cells. The plasticity between T_FH_ and T_FR_ cells have been shown in the same study suggesting that T_FR_ cells may play important roles in suppressing TH2 responses and allergen specific IgE production during immunotherapy (99). It is likely that similar T_FR_ and T_FH_ cell mechanisms are present during venom imunotherapy as in grass pollen immunotherapy.

Recently IL-10-secreting allergen-specific Breg cells have been identified in bee venom tolerant beekeepers and VIT administered patients (100). Breg cells are characterized as CD73^−^ CD25^+^CD71^+^ B cells, which are capable of suppressing bee venom specific CD4^+^ T cells and capable of producing allergen-specific IgG4 antibodies after bee VIT (100). Additionally, Breg cells can also show their inhibitory capacity by producing IL-35 and TGF-beta (101). Apart from Treg and B reg cells, IL-10-secreting natural killer regulatory cells have also been shown to suppress allergen stimulated T cell proliferation in humans and may be important in tolerance induction as other regulatory cell types (102).

### Innate Lymphoid Cells and Allergen Immunotherapy

The effect of allergen immunotherapy on innate lymphoid cells, ILC type 2, has been studied in grass pollen allergy in peripheral blood. AIT supressed seasonal increases in ILC2s in patients treated with immunotherapy compared to untreated controls (103). The decrease in ILC2s correlated with the self reported symptoms. Moreover, the proportion of IL-13^+^ ILC2s also decreased. In another study of seasonal asthmatic patients, Lombardi et al could not show any change in the number of ILC2s during immunotherapy which was explained by non-seasonal measurements while patients were asymptomatic (104). Up to date, there is no evidence that immunotherapy has any effect on epithelially derived cytokines, such as IL-25, IL-33, and TSLP which have regulatory effects on local type 2 inflammation and ILCs (54).

### Histamine and Histamine Receptors on VIT

During VIT an early desensitization develops within days or even hours depending on the type of immunotherapy protocol used, such as rush and ultrarush type of VIT. There is a decrease in basophil numbers, preformed mediators and mediator release by time (75, 105, 106). Among four different type of histamine receptors histamine receptor type 2 (HR2) plays important roles with the peripheral antigen tolerance (89). Basophil supression starts by the activation of histamine type 2 receptors (HR2). H2R decreases allergen-induced FceRI-mediated basophil degranulation and mediator release (107). HR2 is mainly involved in tolerogenic immune responses. It is upregulated in Th2 cells and both suppress allergen stimulated T cell responses and increase IL-10 production in beekeepers (89) which induces the development of peripheral tolerance (76, 108, 109). Histamin via HR2, induces IL-10 production by dendritic cells and Th2 cells (110); increases the suppressive effect of TGF-b on T cells (111) and decreases IL-4 and IL-13 production whicch are the main Th2 type cytokines (112).

## Efficacy and Safety of Venom Immunotherapy

The efficacy of VIT ranges from 77 to 84% for honeybee and from 91 to 96% for wasp venom (9–11). Some factors, such as higher amount of allergen transferred during each sting reaction, consistency of the honeybee sting, diversity of the sensitization pattern of honeybee venom are among the proposed factors which may explain the lower success rates related with the honeybee VIT (9). VIT was found to be effective even after the build up phase and in one study with honebee venom a success rate of 89% was reported with sting challenge 1 week after reaching maintenance dose (113).

Considering honeybee venom immunotherapy, usage of component resolved sensitization patterns may help to increase the treatment success. Some patients are sensitized predominantly to Api m 10, which is an underrepresented allergen in some VIT preparations that may cause a treatment failure (114, 115).

During the build-up phase of VIT, if SSR is a problem to reach the maintenance dose, premedication with omalizumab, an anti-IgE antibody, may be an option (116).

The dose of venom used during VIT is also important to prevent treatment failure. Usually a maintenance dose of 100 μg venom during VIT is sufficient for protection (9). When the risk factors are high, as in beekeepers, a higher dose of venom gives better results (117). If a systemic reaction develops following a field sting or sting challenge during a conventional dose of 100 μg, a higher dose, 200 μg, is recommended (118).

Relapse rates up to 10% has been reported in 1–5 years after stopping of vespula VIT and more common in honeybee VIT (9). In one study a relapse rate of 7.5% for vespula VIT and 15.8% for honeybee VIT were reported 3–5 years after stopping VIT (17). In children, VIT shows a better prognosis compared to adults and only 5% of the children develop moderate to severe systemic reactions to stings up to 20 years of follow-up after discontinuing VIT (15).

Duration of the VIT is important for the efficacy. One year of treatment has failed in nearly one quarter of patients when they stung in 3–4 years after VIT (119). Studies of at least 5 years of treatment seem to show better protection compared to 3 years of treatment (120). Lerch et al showed lower systemic reaction rates in patients receiving VIT for ≥50 months compared to those treated for 33–49 months (5 vs. 18%, respectively) (17). In another study, Golden et al. evaluated patients treated with VIT for at least 5 years and found a systemic reaction rate of 9.5% within 5 years after discontinuing VIT (121).

A recent meta-analysis including five systematic reviews, five RCTs, three controlled (before and after) studies and four case series, showed that VIT significantly reduced the risk of severe systemic reactions (OR = 0.08, 95%CI 0.03–0.26); improved quality of life (risk differenece: 1.41, 95%CI 1.04–1.79) and may be cost effective in those who experienced repeated systemic reactions and impaired quality of life (122).

Adverse events are usually mild during VIT. Patients having systemic reactions develop relapse much more frequently compared to those who did not (16.4–38% vs. 5.4–8%) (11).

Some of the previous risk factors for systemic adverse events during VIT are no longer considered as important risk factors. These older risk factors include mastocytosis, ACE inhibitors, beta-blocker use, high specific IgE levels and skin prick test positivity at low test concerntrations (9). High basal tryptase levels in vespid allergy may be a risk factor for systemic adverse events in VIT (12) but not in honeybee venom allergy (123).

Risk factors for relapse of SSR after stopping VIT are given in Table 2.

**Table 2:** Risk factors for relapse of severe systemic reactions after stopping VIT (9, 11, 15, 124–127).

**RISK FACTOR**	
Honeybee VIT (9)	Higher risk compared to vespid VIT
Systemic adverse events during VIT (125, 128)	A significant increase in risk of relapse
Severe reaction prior to VIT??	Contoversial but greater risk for severe systemic reactions when relapse
Mastocytosis/high serum tryptase??	Conflicting results but not seem to be an important risk factor. If there is severe initial systemic reaction may be considered as a risk factor
Angiotensin converting enzyme inhibitors??	Conflicting results. The risk of relapse may be overestimated because of the small sample size and highly selected patient groups

Currently, sting challenge is the gold standard to identify the efficacy of VIT and to differentiate the responders from non-responders. The methods used to monitor VIT are given in Table 3.

**Table 3:** The methods used to monitor Venom immunotherapy (VIT) (9, 17, 74, 125, 129).

**METHOD**	
Sting challenge/Field sting	Sting challenge is the Gold Standard. If not possible field stings may be used. However, field stings are not standardized and some difficulties to identify the stinging insect type
SIgE and IgG4	sIgE serum levels decreases and IgG4 levels increase during VIT. However, protection from systemic reactions continues, although IgG4 decreases after VIT is discontinued. Their levels and ratios are not reliable to predict individual protection
Intradermal skin prick testing	This method is not predictive as the negative skin prick test result can not exclude a relapse wih a serious systemic reaction
Basophil sensitivity	The dose at which half of the maximum basophil response occurs, was suggested to monitor VIT
Enzyme-linked immunosorbent facilitated antigen binding	Only case series studies are present. Inhibitor activity decreases after stopping VIT. Not possible to estimate the individual risk of relapse

## Conclusion

Insect venom allergy is one of the most common causes of anaphylaxis in humans and it is a medical emergency. Currently there is no biomarker to predict the risk of anaphylaxis. VIT is the most effective treatment for preventing SSR to a sting and decreases the risk of anaphylaxis. However, there are still some questions to be answered, such as cost of effectiveness, effect on quality of life, duration of treatment, optimal dose and means of assessment. Peripheral tolerance development is the main mechanism during VIT, which is orchestrated by T regulatory cells. T regs produce IL-10 and suppress Th2 immunity and the immune responses shift toward Th1 type inflammation. Blocking IgG1/IgG4 antibodies inhibit IgE dependent reactions on mast cells, basophils and B cells. Several other mechanisms, such as epithelial cells, several cytokines, dendritic cells, ILC2s and B regulatory cells are also involved in the development of long term immune tolerance.

Factors, such as increasing knowledge about risk factors for venom anaphylaxis, better patient education, developing more effective VIT products with less side effects, and finding effective biomarkers to predict future systemic reactions at the individual level, will significantly improve patient care.

## Author Contributions

US made the literature search, prepared the figure and tables, and wrote the article under the supervision of SD. SD planned the whole body of text, read the manuscript, made the necessary corrections, and detailed the discussion on the mechanisms of immunotherapy.

### Conflict of Interest Statement

SD reports grants from ALK, Denmark, personal fees from Anergis, Switzerland, personal fees from Biomay, Austria, personal fees from Allergy Therapeutics, UK, personal fees from ALK, Horsholm, Denmark, personal fees from Allergy Therapeutics, outside the submitted work. The remaining author declares that the research was conducted in the absence of any commercial or financial relationships that could be construed as a potential conflict of interest.
